# Study on an Epoxy Resin System Used to Improve the Elasticity of Oil-Well Cement-Based Composites

**DOI:** 10.3390/ma15155258

**Published:** 2022-07-29

**Authors:** Jianjian Song, Mingbiao Xu, Chunqin Tan, Fuchang You, Xiaoliang Wang, Shanshan Zhou

**Affiliations:** 1 Cooperative Innovation Center of Unconventional Oil and Gas, Ministry of Education & Hubei Province, Yangtze University, Wuhan 430100, China; songjian629@yangtzeu.edu.cn (J.S.); yfc81@163.com (F.Y.); wangxiaoliang031@163.com (X.W.); 2 School of Petroleum Engineering, Yangtze University, Wuhan 430100, China; 2021730007@yangtzeu.edu.cn; 3 Key Laboratory of Drilling and Production Engineering for Oil and Gas, Hubei Province, Wuhan 430100, China; 4 Sinopec Petroleum Engineering Technology Research Institute, Beijing 100101, China; tancq.sripe@sinopec.com

**Keywords:** epoxy resin, oil-well cement, cementing, mechanical properties, structural characteristics

## Abstract

Oil-well cement-based materials have inherent brittleness; therefore, they cannot be directly used to seal oil and gas wells for a long time. To improve the elasticity of oil-well cement-based composites, a flexible epoxy resin system was developed. The flexibility, TG, and SEM of the cured resin system were evaluated. At the same time, the resin was added to oil-well cement-based materials to improve its elasticity. The compressive strength and elastic modulus of resin cement stone were tested, and the microstructure was analyzed by XRD, TG, and SEM/EDS. The results showed that the structure of the cured resin is compact, the thermal decomposition temperature is 243.9 °C, and it can recover its original shape after compression. At the curing age of 28 days, the compressive strength of cement-based composites containing 30% resin decreased by 26.7%, while the elastic modulus significantly decreased by 63.2%, and the elasticity of cement-based composites was significantly improved. The formation of hydration products (e.g., calcium silicate hydrate, and calcium hydroxide) in the resin cement slurry is obviously lower than that of pure cement, which is the reason for the decrease in compressive strength. The flexible structure of polymer particles and polymer film formed by epoxy resin is distributed inside the cement stone, which significantly improves the elasticity of oil-well cement-based composites. The results of this paper are helpful for the design of elastic cement slurry systems.

## 1. Introduction

Oil- and gas-well cementing is a process of injecting cement slurry into the annulus to realize interlayer sealing, and to support and protect casing. With the extension of oil- and gas-well development time, the development problems caused by seal failure are becoming more and more serious, and the requirements for the performance of cement stone are also increasing [1,2,3]. Shale gas, tight gas, and other wells with complex stress require that the materials used to seal the wellbore have high elasticity to ensure the long-term effectiveness of the cement’s sheath. However, the unmodified oil-well cement is a brittle material, which cannot realize the safe and efficient sealing of complex oil and gas wells.

Currently, the primary materials used in oil-well cement to improve the elasticity of cement paste are fiber, latex and elastic particles. Fibers mainly include inorganic and organic fibers, which can improve the internal bonding performance of cement pastes by “bridging” and limit the development of cracks when the cement paste is damaged [4,5]. Latex can form a continuous polymer membrane structure in oil-well cement, which can improve the flexibility and toughness of cement paste at the same time [6]. The elastic particles are mainly rubber powder, which is filled in the cement stone. When the cement stone is compressed, the elastic particles form a flexible structure center to improve the elasticity of the cement stone [7]. These materials are widely used in oil-well cement, but the amount of these materials cannot be too much as it can easily have adverse impacts on oil-well cement. For example, having too many fibers will cause twisting, latex is unstable in the high shear state, and the hydrophilicity of elastic particles is poor. It is necessary to study the highly flexible and modified materials that can be added to oil-well cement with large dosages to improve the elasticity of cement paste.

Epoxy resin is a kind of flexible polymer material that can form elastomers with hardeners. After adding epoxy resin and a curing agent to cement-based materials, the mechanical properties of the cement slurry are more effectively improved than that without a curing agent [8,9]. Epoxy resin containing curing agents is widely used in building materials, but the application environments of oil-well cement are complex, often with high temperature and pressure. The complex application environment does not permit the direct use of epoxy resin systems, that used in building materials, in oil-well cement. The application of resin-containing curing agents in oil-well cement-based materials is limited, mainly because the thickening time of resin–cement-based composites is not easy to control, and its compatibility is poor. Du et al. [10], A. Yami et al. [11], and Liu et al. [12] designed resin cement slurry and mixed resin into cement slurry. The elastic modulus of resin-modified cement slurry is lower than that of non-resin cement slurry. In addition, studies on the microstructures and interfacial properties of resin-modified cement-based composites by A.R. Cestari et al. [13] and F. Djouani et al. [14] showed that the addition of resin into cement slurry influences the structural characteristics of cement paste; the resin can be evenly mixed with cement-based materials. However, most of the existing studies focus on resin cement-based composites, and there is no systematic report on the performance and flexibility of the resin system used in oil-well cement. Moreover, according to the micromechanical model of the composite, the properties of the resin are closely related to the mechanical properties of the composite [15]. Generally speaking, the greater the flexibility of the resin, the higher the elasticity of the resin cement-based composites. The study of epoxy resin systems with high flexibility is of great significance for the design of highly elastic resin cement slurry.

In this work, an epoxy resin was synthesized in the laboratory to design a flexible cement slurry system. The prepared resin system has good flexibility, and it can be mixed with oil-well cement in a large dosage to improve the elasticity of oil-well cement. The modified resin–cement stone has an excellent elastic modulus. The macroproperties and microstructures of the prepared resin and resin cement slurry are evaluated and discussed, and the mechanism of the prepared elastic resin in oil-well cement is analyzed. Research results will provide technical support for oil and gas well cementing.

## 2. Experiment and Method

### 2.1. Materials

Oil-well cement (class G Portland cement) was procured from the Jiahua Special Cement Co., Ltd., Leshan, China. Micro silica and hardener were obtained from the Jingzhou Jiahua Technology Co., Ltd., Jingzhou, China. Micro silicon is mainly used to improve the strength and stability of cement slurry. Filtration reducer and dispersant were prepared in the laboratory, and were used to reduce the water loss of cement slurry and improve the fluidity of cement slurry, respectively.

The epoxy resin was synthesized and produced in the laboratory. The production methods were as follows: The epoxy resin precursor was obtained by mixing propylene oxide butyl ether, butyl acrylate, resorcinol and demonized water and reacting at 80 °C for 4 h. The mixing ratio of propylene oxide butyl ether, butyl acrylate, resorcinol, and purified water is 15:20:35:30. Then, the precursor and a 30% alkaline solution were mixed and reacted for 3 h at 80 °C. The ratio of the precursor and alkaline solution is 5:2. Consecutively, solid epoxy resins were obtained by washing and drying the reaction product. Finally, the solid resin product was mixed with the active diluent at the proportion of 1:1 and stirred at a speed of 300 r/min for 5 h at 60 °C. 

### 2.2. Specimen Preparation

**Figure 1:**
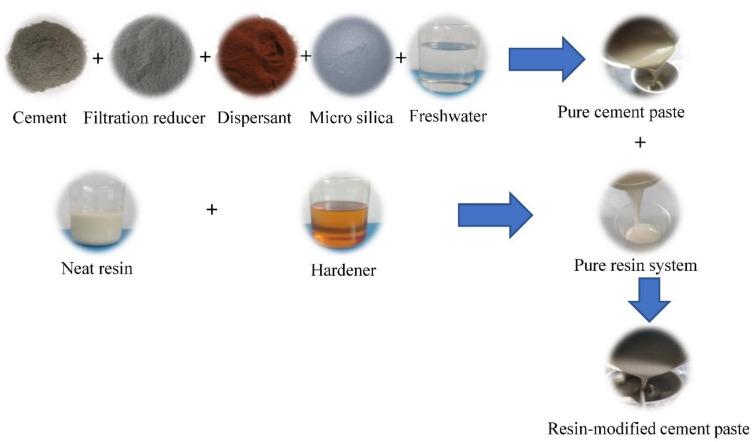
Sample preparation procedures.

The neat resin and hardener were weighed by an electronic balance according to the specific compositions of the pure resin system, as shown in Table 1. The hardener was added to the neat resin and mixed uniformly as a pure resin system. Second, according to the components in Table 2, the cement, filtration reducer, microsilica, and dispersant were weighed as dry powder, fresh water was added in quantity, and a constant speed agitator (TG-3060, Shenyang Taige Oil Equipment Co., Ltd., Shenyang, China) was used to mix the dry powder and fresh water as cement paste at a speed about 4000 r/min. Subsequently, the pure resin system and the cement slurry system were mixed and stirred to form the resin-modified cement-slurry system according to the volume proportion in Table 3. The sample preparation procedures are shown in Figure 1.

### 2.3. Thermogravimetric (TG) Analysis

A thermogravimetric (TG) analysis of the cured pure resin system was performed with a synchronous thermogravimetric analyzer (STA449F5, Netzsch, Selb, Germany). In this experiment, the test environment was full of nitrogen, and the temperature increased from 25 °C to 600 °C at a rate of 10 °C/min.

### 2.4. Flexibility of the Pure Resin System

After the preparation of the resin system, it was poured into a cube mould (50.8 × 50.8 × 50.8 mm^3^) and then cured at 80 °C for 24 h. Then, the compression of the cured resin was tested by a mechanical testing machine (HY-20080, Shanghai Hengyi Precision Instrument Co., Ltd., Shanghai, China) at a constant speed of 8 mm/min. The compression degree of the resin specimen was judged by the compression displacement during testing. The deformation recovery of the resin sample was measured after the loading was removed. The compression degree *V* and deformation recovery *H* were calculated according to the following formula:

(1) $$V=\frac{L_{0}}{L}$$

(2) $$H=\frac{L_{1}}{L}$$

where *L*_0_ is the compression displacement during testing, and *L*_1_ is the length (along the compression direction) of the sample after recovery. *L* is the size of the original specimen and equal to 50.8 mm. 

### 2.5. Mechanical Properties

The prepared resin-modified cement slurry was poured into the mould and maintained for different lengths of time at 80 °C in the water bath at atmospheric pressure. When the specified curing time was reached, the mould was removed and then the solidified cement samples were wiped clean. The sample could be tested after reaching room temperature. The uniaxial compressive strength and modulus of elasticity of the cement paste were tested by the mechanical testing machine according to the Chinese standard GB/T 50266-99 at a constant loading rate of 400 N/s. The size of the tested specimen was 50.8 × 50.8 × 50.8 mm^3^, and three samples were tested to calculate the average value.

### 2.6. X-ray Diffraction (XRD) 

X-ray diffraction (XRD) was performed to analyse the phase composition of the hydration products of the cement paste using an X-ray diffractometer (D8 Advance, Bruker, Karlsruhe, Germany). The test range of 2θ is 10–70° with Cu Kα radiation. 

### 2.7. Scanning Electron Microscopy/Energy Dispersive Spectrometer (SEM/EDS)

After preparing the cement sample, the small flat pieces inside the cement stone may be taken for surface gold plating. The micromorphology and characteristics of the pure resin system and resin-modified cement stone were observed by scanning electron microscopy (SEM) (SU8010, Hitachi, Tokyo, Japan) in high vacuum mode. The elements of the sample were analyzed by the energy dispersive spectrometer (EDS) (Ultim Max 40, Oxford, Oxford, UK) equipped with the scanning electron microscope.

## 3. Results and Discussion

### 3.1. Properties of Cured Resin System

#### 3.1.1. Flexibility of Cured Resin System

**Figure 2:**
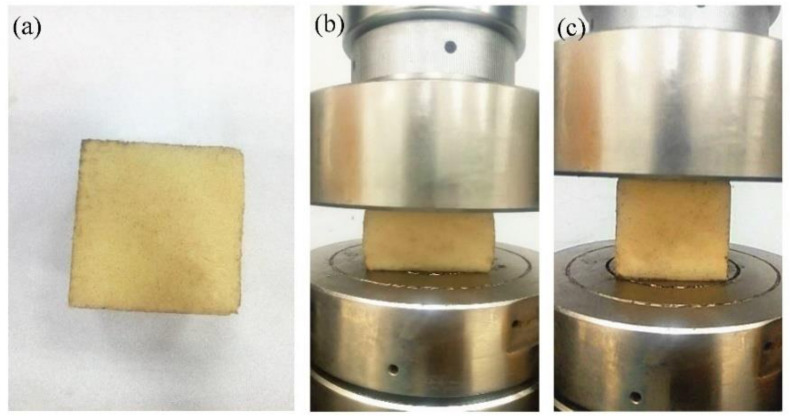
Testing process of compression and shape recovery ((**a**). original sample; (**b**). compressed by 50%; (**c**). deformation recovery).

The developed resin system was a complete elastomer after curing. The flexibility of the resin system was evaluated before its application in oil-well cement. According to Formulas (1) and (2), the deformation recovery (H) of the resin under 50% compressive deformation with different curing times was tested, and the test results are shown in Table 4. The testing process of compression and shape recovery is illustrated in Figure 2. The data in Table 4 show that the resin sample can still recover its original shape under different compression conditions, and the recovery ratio of deformation is close to 100%. This indicates that the cured resin has excellent flexibility, which helps improve the elasticity of the composite. In Figure 2, the resin is intact during a state of heavy compression, and the original shape can be recovered after the external force is unloaded. The results show that the epoxy resin has high deformability after curing.

#### 3.1.2. Thermogravimetric Analysis

The materials used for oil and gas well sealing are often subjected to certain temperatures; therefore, it is necessary to study the thermal stability of the resin system. The TG test is a thermal analysis technique used to measure the relationship between weight and temperature variations under programmed temperature control; the latter is utilized in this process to study the thermal stability of materials [16,17]. Under the thermal decomposition temperature, the resin can maintain its service life for a long period of time [18].

**Figure 3:**
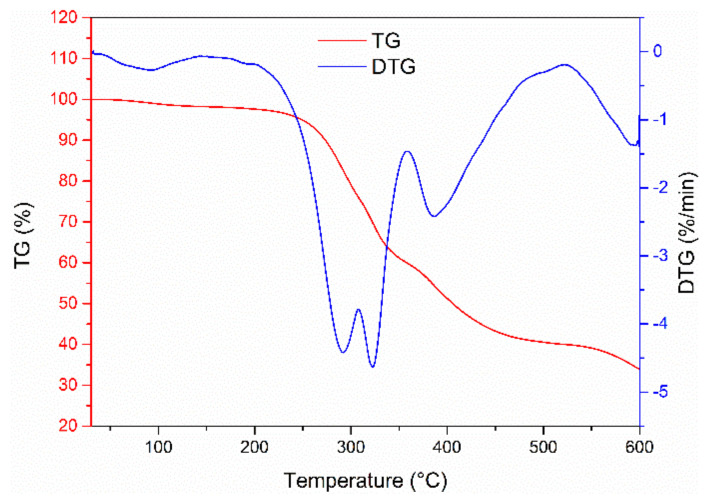
TG-DTG curve of the cured resin system.

Figure 3 shows that the thermogravimetric loss process of cured resin at different temperatures can be divided into three stages. In the first stage, the temperature ranges from 25 °C to 243.9 °C, and the percentage of thermogravimetric loss of the resin is 4.4%. The variation of thermogravimetric loss rate is irregular and modest. In the second stage, the temperature range is 243.9~522.6 °C, and the percentage of thermogravimetric loss is 55.6%. The thermogravimetric loss rate first increases rapidly and then changes with increasing temperature. The maximum weight loss rate is 4.6%/min. In the last stage, the temperature ranges from 522.6 °C to 600 °C, and the thermogravimetric loss is approximately 6%. The change in the thermogravimetric loss rate is irregular. When the temperature reaches 600 °C, the total weight loss of the sample is 66%, and the residual weight of the sample is 34%. The experimental results show that the critical thermal decomposition temperature of the resin is 243.9 °C. The temperature of resin used as a modifier in oil-well cement slurry should be below the critical temperature to ensure the function of the resin in oil-well cement.

#### 3.1.3. Micromorphology of Cured Resin System

**Figure 4:**
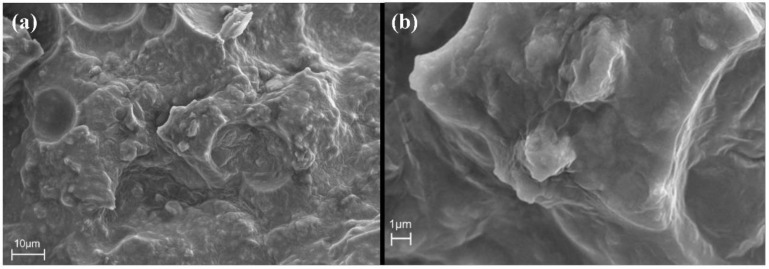
SEM images of cured resin system ((**a**). low magnification; (**b**). high magnification).

The micromorphology of the material can be observed in SEM images. Figure 4a,b show low magnification and high magnification pictures of the cured resin system, respectively. Figure 4a shows that the internal structure of the resin is coarse; there are many gel particle clusters on the entire observation surface, and the size of the gel particles varies. Figure 4b shows that there is no pore or fracture structure in the resin, and the gel structure is dense. The structure of the resin is conducive to the formation of defect-free cement-based composites.

### 3.2. Mechanical Properties of Resin-Modified Cement-Based Composites

The purpose of developing an epoxy resin system is to improve the elasticity of oil-well cement-based composites. Therefore, when evaluating the effect of a resin system on the mechanical properties of oil-well cement, the compressive strength and elastic modulus of the resin-modified cement-based composites are measured. The compressive strength must resist the horizontal compressive force of formation pressure on cement paste and the vertical tension caused by the casing [19,20]. Elastic modulus is a physical parameter that describes the ability of solid materials to resist deformation, and its value is the ratio of stress to strain [21]: The greater the value, the worse the elasticity. A low elastic modulus is more helpful for ensuring the integrity of the cementing cement sheath.

**Figure 5:**
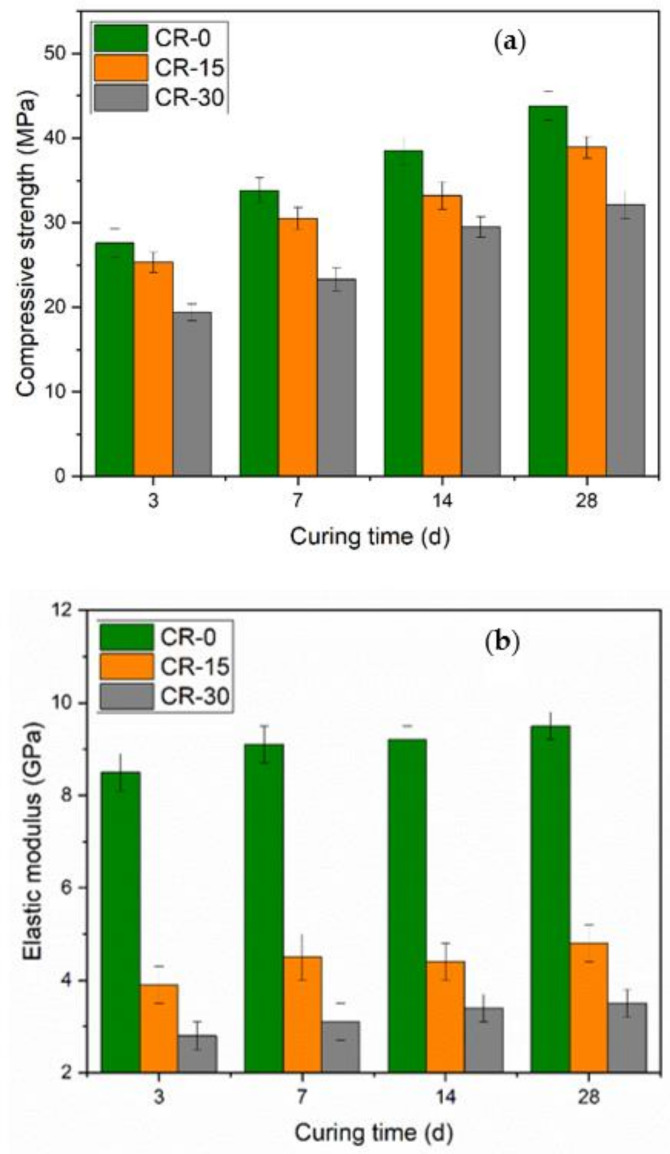
Mechanical properties of resin-modified cement-based composites ((**a**). compressive strength; (**b**). elastic modulus).

Figure 5a shows the compressive strength of the resin-modified cement slurry at different curing times. When the curing time is extended, the compressive strength of cement pastes increases. In the initial stage, the compressive strength increases rapidly. Resin has an influence on the compressive strength of cement paste: When more resin is added to the mixture, the compressive strength of the cement paste lowers. The compressive strength of pure cement paste (CR-0) reached 27.6 MPa after curing 3 days. The compressive strength of oil-well cement-based composites samples containing 15% (CR-15) and 30% (CR-30) resin was reduced by approximately 8.3% and 29.7%, respectively, compared with the CR-0 sample. At the curing age of 28 days, the compressive strength of the pure cement paste was 43.8 MPa, while the compressive strength of resin-modified cement-based composites CR-15 and CR-30 decreased by 11.2% and 26.7%, respectively, compared with the CR-0 sample.

Figure 5b shows the modulus of elasticity of the resin-modified cement-based composites. The longer the curing time, the larger the elastic modulus of the cement paste. The increase in the elastic modulus of the cement paste was notable within 7 days. When cured for 3 days, the elastic modulus of the cement paste was 8.5 GPa. The elastic modulus of cement paste was 11.8% higher than that of 3 days after curing for 28 days. With increasing resin content, the modulus of elasticity of the cement paste decreased significantly. Compared with non-resin cement-based composites, the elastic modulus of CR-15 and CR-30 samples was reduced by 49.5% and 63.2%, respectively, when the curing age was 28 days.

Considering the decreasing range of compressive strengths, the addition of the resin system improved the deformation ability and reduced the elastic modulus of the cement-based composites more significantly. For CR-30, the compressive strength decreased by 26.7%, while the elastic modulus decreased by 63.2% after curing for 28 days. Since the elastic modulus is the ratio of stress and strain, it can be considered that the decrease in elastic modulus of cement stone is mainly caused by the increase in deformation capacity. This is also characteristic of the flexibility of cement stone.

### 3.3. Structural Characteristics of Resin-Modified Cement-Based Composites

#### 3.3.1. Phase Analysis

The setting and hardening of cement are complex physical–chemical processes. When cement minerals are mixed with water, hydration reactions occur to form hydration products. These hydration products overlap and connect with each other by various gravitational forces in a certain way to form the structure of the cement paste, resulting in strength [22,23]. When a resin system is added to oil-well cement, the presence of an organic component may affect the hydration products or the state of the products.

**Figure 6:**
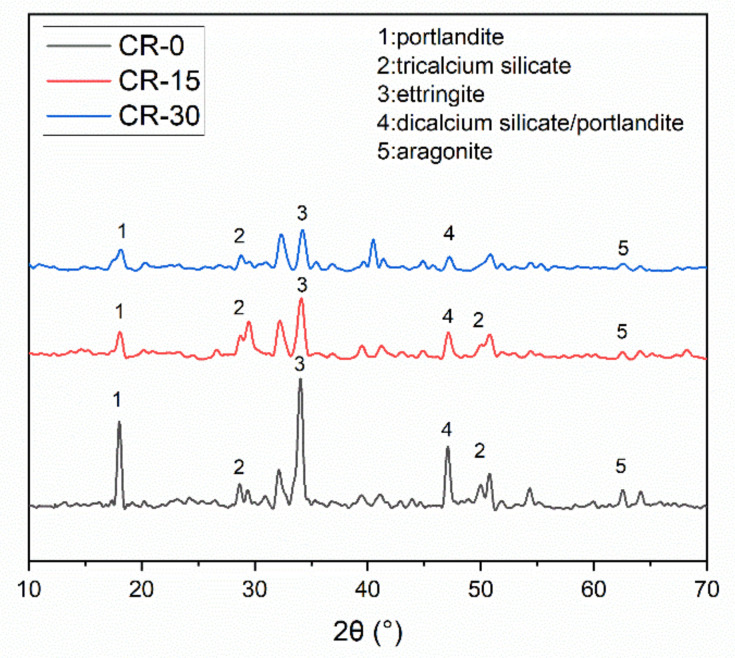
XRD patterns of resin-modified cement slurry.

The XRD patterns of the resin-modified cement paste are shown in Figure 6. For the pure cement slurry (CR-0), the XRD analysis results indicate that the hydration products mainly include: portlandite (2θ = 18°); tricalcium silicate (C_3_S, 3CaO·SiO_2_) (2θ = 28°,51°); ettringite (2θ = 34°); a mixture of dicalcium silicate [C_2_S, 2CaO·SiO_2_] and portlandite (2θ = 47°); and aragonite (CaCO_3_) (2θ = 63°). For the cement slurries containing 15% resin (CR-15) and 30% resin (CR-30), their hydration products are mainly calcium silicate hydrates, ettringite, and portlandite. No extra hydration products were generated, as opposed to pure cement slurry. Overall, the characteristic peak of the XRD curve of the non-resin cement paste is clearly stronger than that of the resin-modified cement-based composites. This observation indicates that the addition of resin reduces the formation of hydration products, particularly calcium silicate hydrates (CSH) (which provides strength for the cement paste) [9,13]. It is the reason for the decrease in compressive strength.

#### 3.3.2. Thermogravimetric Analysis

The main hydration products of oil-well cement are hydrated calcium silicate, hydrated calcium sulphoaluminate, and calcium hydroxide. As the resin is mixed into oil-well cement to form composite materials, the resin will be decomposed with the increase in temperature. Therefore, the loss of modified cement slurry with different resin content has been analyzed.

**Figure 7:**
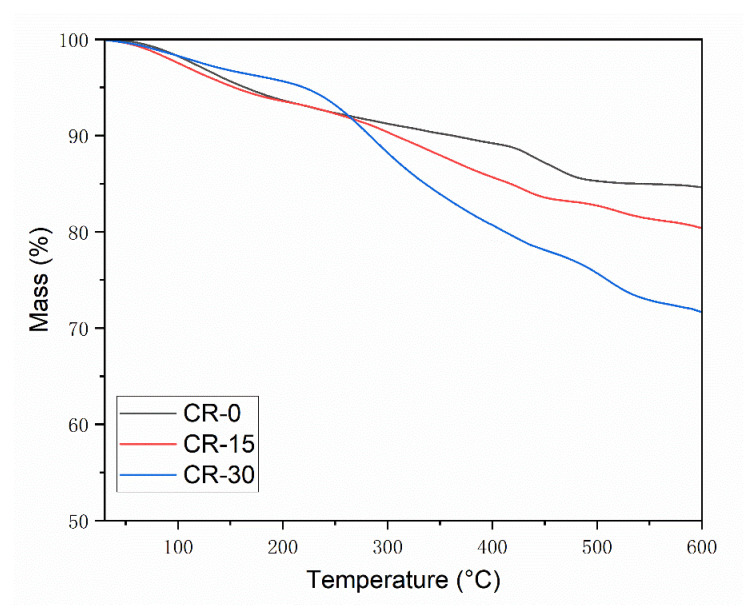
Thermal analysis curves of different cement samples.

Figure 7 shows the results of the thermogravimetric analysis of cement paste. The mass loss of different samples increases with the increase in temperature. When the temperature reached 600 ℃, the mass loss of CR-0, CR-15, and CR-30 reached 15.4%, 19.6%, and 28.3%, respectively. The higher the resin content, the greater the mass loss of the cement paste. In the early stage, the mass loss of different samples was modest. When the temperature exceeded 243.9 °C, the mass loss of the resin-modified cement-based composites increased rapidly, which is consistent with the mass loss of the pure resin sample. However, the mass loss of resin is reduced when the cement is combined with the resin. At the same time, calcium hydroxide will be decomposed at 420–510 °C [24]. The figure shows that the decomposition curve of resin-free samples at 420–510 °C has a sharp decline, while other samples do not share this phenomenon. It shows that the content of calcium hydroxide in pure cement samples is much higher than that of the resin cement samples. The hydration degree of pure cement is higher than that of resin-the modified cement-based composites.

#### 3.3.3. Micromorphology Analysis

**Figure 8:**
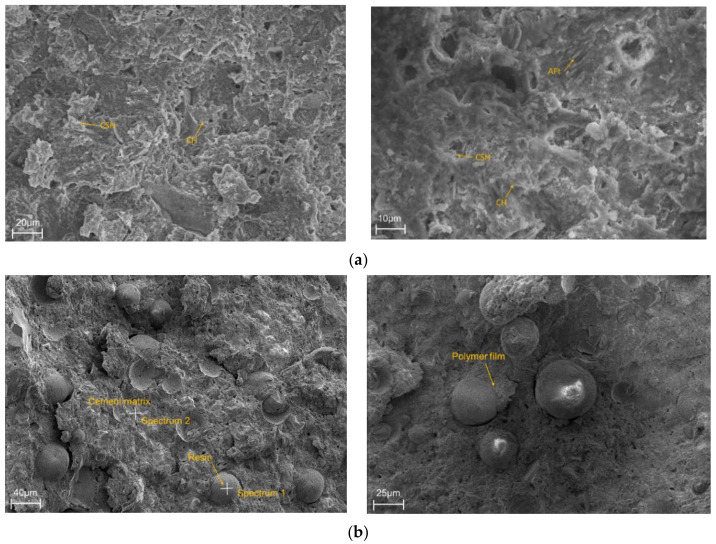
Micromorphology of pure cement slurry and resin cement slurry. (**a**) Micromorphology of CR-0; (**b**) Micromorphology of CR-30.

Figure 8 shows the micromorphology of the pure cement slurry CR-0 and resin cement slurry CR-30. For sample CR-0, rod-shaped ettringite (AFt), lamellar calcium hydroxide (CH), and fibrous calcium silicate hydrate (CSH) can be seen in the SEM photos. The high magnification SEM image shows that the cement hydration products are clear, and the structure is obvious. The fibrous CSH forms a reticular skeleton inside the cement paste, which is the main product supporting the strength of the cement paste. For resin cement slurry CR-30, the low-power photos show the resin particles filled in the cement hydration products, and this polymer structure is firmly combined with the cement hydration products. The high-power photo shows the same microstructure as that of the cured resin around the resin particles in the sample, and a similar membrane-like material formed to cover the surface of the hydration product, which reduces the compressive strength and greatly improves the elasticity of the cement stone.

EDS analysis of the sample can explain the elementary composition of hydrate products [25]. Figure 8 shows the EDS results of the resin-modified cement paste CR-30. Table 5 and Table 6 list the element contents of resin (spectrum 1) and cement matrix (spectrum 2), respectively. The location of the EDS test is shown in Figure 8b. From the analysis of Table 5, the region of spectrum 1 contains a high content of C, which can be determined as resin particle. The main elements in the resin matrix are C and O, and the weight and atomic percentage of the two elements account for 91.58% and 97.14% of all elements. Among them, the weight ratio of the carbon element is the highest, reaching 67.83%. According to the elementary analysis shown in spectrum 2 in Table 6, the high content of Ca indicates that the region of spectrum 2 consists of a cement matrix formed by hydration products. The results show that the main elements in the cement matrix are Ca, O, C, and Si. The weight and atomic percentage of these four elements account for 87.38% and 92.06% of all elements. Among them, the weight ratio of calcium is the highest, reaching 39.17%. Calcium and carbon were selected because they are specific elemental markers for the mineral and the polymer, respectively. The results of EDS analysis showed the position of resin polymer particles in the micromorphology of cement-based composites.

## 4. Conclusions

In this paper, a highly flexible resin was prepared, and its flexibility, thermal analysis, and micromorphology after curing were evaluated. At the same time, the resin was added to oil-well cement-based composites, and its mechanical properties and microstructure were analyzed. These studies have reached the following conclusions:

(1) The structure of cured resin is compact, and its thermal decomposition temperature is above 243.9 °C, which is higher than the application temperature of cement slurry. The shape recovery rate of cured resin elastomer is nearly 100% after being subjected to large compressive strain.
(2) After the resin system mixed with oil-well cement, the compressive strength and elastic modulus of cement stone containing 30% resin decreased by 26.7% and 63.2%, respectively, compared with the blank cement stone. The decrease in elastic modulus is much larger than that of the compressive strength, and the elasticity of the cement stone obviously improved.
(3) The addition of resin delays the hydration of cement and reduces the formation of hydration products, which is the reason for the decrease in compressive strength.
(4) Due to the decomposition of resin, the mass loss of resin cement-based composites increases when the temperature is greater than 243.9 °C, and due to increased calcium hydroxide, the pure cement stone decreases sharply at 420–510 °C.
(5) In the hydration products filled with resin particles, the morphology is consistent with that of the cured resin in the vicinity of the resin particles, and the hydration products are covered with polymer film. These effects reduce the elastic modulus of the cement-based composites.

## Figures and Tables

**Table 1:** Pure resin system components.

Material	Content (wt%)
Neat resin	100
Hardener	20

**Table 2:** Cement paste components.

Material	Content (wt%)
Cement	100
Freshwater	44
Filtration reducer	2
Dispersant	1
Micro silica	1.5

**Table 3:** Volume proportion of resin and cement slurry in resin-modified cement paste.

Sample Number	Cement Slurry Content (%)	Resin System Content (%)
CR-0	100	0
CR-15	85	15
CR-30	70	30

**Table 4:** Deformation recovery of resin system under 50% compressive deformation.

Curing Time (d)	V (%)	H (%)
3	50.0	100
7	50.0	98.6
14	50.0	99.2
28	50.0	100

**Table 5:** Element content of the resin (Spectrum 1).

Element	wt%	Atomic%
C	67.83	76.69
O	23.75	20.45
Si	0.33	0.16
S	0.39	0.17
Cl	2.35	0.81
Ca	4.28	1.58
Zr	1.07	0.14
Total	100.00	100.00

**Table 6:** Element content of the cement matrix (Spectrum 2).

Element	wt%	Atomic%
C	15.02	28.06
O	25.82	36.22
Na	1.00	0.98
Mg	0.20	0.18
Al	0.92	0.76
Si	7.37	5.88
S	2.67	1.87
Cl	3.86	2.44
K	0.55	0.32
Ca	39.17	21.92
Fe	3.42	1.37
Total	100.00	100.00

## Data Availability

Not applicable.

## References

[1] Yang Y., Deng Y. (2018). Mechanical properties of hybrid short fibers reinforced oil well cement by polyester fiber and calcium carbonate whisker. Constr. Build. Mater..

[2] Jafariesfad N., Sangesland S., Gawel K., Torsæter M. (2020). New materials and technologies for life-lasting cement sheath: A review of recent advances. SPE Drill. Complet..

[3] Li Y., Dong S.F., Ahmed R., Zhang L., Han B. (2020). Improving the mechanical characteristics of well cement using botryoid hybrid nano-carbon materials with proper dispersion. Constr. Build. Mater..

[4] Paiva L.C.M., Ferreira I.M., Martinelli A.E., Freitas J.C.D.O., Bezerra U.T. (2018). Milled basalt fiber reinforced Portland slurries for oil well applications. J. Pet. Sci. Eng..

[5] Zhu J., Wei J., Yu Q., Xu M., Luo Y. (2020). Hybrid effect of wollastonite fiber and carbon fiber on the mechanical properties of oil well cement pastes. Adv. Mater. Sci. Eng..

[6] Zhao J., Hu M., Liu W., Feng J., Zhang H., Liu M., Guo J. (2022). Toughening effects of well-dispersed carboxylated styrene-butadiene latex powders on the properties of oil well cement. Constr. Build. Mater..

[7] Cheng X., Chen Z., Gu T., Zeng L., Yao L., Chen Z., Huang K., Zhang Z., Zhang C., Liu K. (2021). Study on the dynamic and static mechanical properties of microsphere rubber powder reinforced oil well cement composites. Constr. Build. Mater..

[8] Pang B., Zhang Y., Liu G. (2018). Study on the effect of waterborne epoxy resins on the performance and microstructure of cement paste. Constr. Build. Mater..

[9] Cestari A.R., Vieira E.F., Tavares A.M., Andrade M.A. (2010). Cement–epoxy/water interfaces—Energetic, thermodynamic, and kinetic parameters by means of heat-conduction microcalorimetry. J. Colloid Interface Sci..

[10] Du J., Bu Y., Shen Z., Hou X., Huang C. (2016). Effects of epoxy resin on the mechanical performance and thickening properties of geopolymer cured at low temperature. Mater. Des..

[11] Yami A., Buwaidi H., Al-Herz A., Mukherjee T.S., Bedford D., Viso R., Hugentobler K. Application of heavy weight cement-resin blend system to prevent cca pressure in saudi arabia deep gas fields. Proceedings of the SPE Oil and Gas India Conference and Exhibition.

[12] Liu Y.L., Li Z.Y., Xue Y.T., Zhou B., Sun H., Su D.H., Sun J.F. (2019). Study on mechanical properties of cementing waterborne epoxy cement. Bull. Chin. Ceram. Soc..

[13] Cestari A.R., Vieira E.F., Silva E.C., Alves F.J., Andrade M.A. (2013). Synthesis, characterization and hydration analysis of a novel epoxy/superplasticizer oilwell cement slurry—Some mechanistic features by solution microcalorimetry. J. Colloid Interface Sci..

[14] Djouani F., Connan C., Chehimi M.M., Benzarti K. (2010). Interfacial chemistry of epoxy adhesives on hydrated cement paste. Surf. Interface Anal..

[15] Yu Y., Han Z., Zhu X.C., Li H.R. (2015). Effect of the pore structure on the elastic modulus of crumb rubber cement mortar. Bull. Chin. Ceram. Soc..

[16] Chaudhary R.G., Ali P., Gandhare N.V., Tanna J.A., Juneja H.D. (2019). Thermal decomposition kinetics of some transition metal coordination polymers of fumaroyl *bis* (paramethoxyphenylcarbamide) using DTG/DTA techniques. Arab. J. Chem..

[17] Xie Q.Y., Chen D.D., Ding Y.W. (2022). Thermogravimetric analysis and its applications in polymer characterization. Acta Polym. Sin..

[18] Al-Yami A., Wagle V., Jimenez W.C., Jones P. (2019). Evaluation of epoxy resin thermal degradation and its effect on preventing sustained casing pressure in oil and gas wells. Arab. J. Sci. Eng..

[19] Song J., Xu M., Liu W., Wang X., Xu P., Huang F., Pan Y. (2019). Thermoplastic rubber (TPR) modified by a silane coupling agent and its influence on the mechanical properties of oil well cement pastes. Adv. Mater. Sci. Eng..

[20] Maagi M.T., Jun G. (2020). Effect of the particle size of nanosilica on early age compressive strength in oil-well cement paste. Constr. Build. Mater..

[21] Ouyang X., Shi C., Wu Z., Li K., Shan B., Shi J. (2020). Experimental investigation and prediction of elastic modulus of ultra-high performance concrete (UHPC) based on its composition. Cem. Concr. Res..

[22] Moghadam H.A., Mirzaei A., Dehghi Z.A. (2020). The relation between porosity, hydration degree and compressive strength of Portland cement pastes in the presence of aluminum chloride additive. Constr. Build. Mater..

[23] Liu Z., Sha A., Hu L., Zou X. (2017). A laboratory study of Portland cement hydration under low temperatures. Road Mater. Pavement Des..

[24] Zhang Z., Du J., Shi M. (2022). Quantitative Analysis of the Calcium Hydroxide Content of EVA-Modified Cement Paste Based on TG-DSC in a Dual Atmosphere. Materials.

[25] Du J., Bu Y., Shen Z. (2018). Interfacial properties and nanostructural characteristics of epoxy resin in cement matrix. Constr. Build. Mater..

